# Insight into the neurophysiological processes of melodically intoned language with functional MRI

**DOI:** 10.1002/brb3.245

**Published:** 2014-07-03

**Authors:** Carolina P Méndez Orellana, Mieke E van de Sandt-Koenderman, Emi Saliasi, Ineke van der Meulen, Simone Klip, Aad van der Lugt, Marion Smits

**Affiliations:** 1 Department of Radiology, Erasmus MC – University Medical Center RotterdamRotterdam, The Netherlands; 2 Department of Neurology, Erasmus MC – University Medical Center RotterdamRotterdam, The Netherlands; 3 Rehabilitation Medicine, Erasmus MC – University Medical Center RotterdamRotterdam, The Netherlands; 4 Rijndam Rehabilitation CenterRotterdam, The Netherlands; 5 Department of Neurology - University Medical Center GroningenGroningen, The Netherlands

**Keywords:** Aphasia, auditory perception, fMRI, language, language therapy, singing

## Abstract

**Background:**

Melodic Intonation Therapy (MIT) uses the melodic elements of speech to improve language production in severe nonfluent aphasia. A crucial element of MIT is the melodically intoned auditory input: the patient listens to the therapist singing a target utterance. Such input of melodically intoned language facilitates production, whereas auditory input of spoken language does not.

**Methods:**

Using a sparse sampling fMRI sequence, we examined the differential auditory processing of spoken and melodically intoned language. Nineteen right-handed healthy volunteers performed an auditory lexical decision task in an event related design consisting of spoken and melodically intoned meaningful and meaningless items. The control conditions consisted of neutral utterances, either melodically intoned or spoken.

**Results:**

Irrespective of whether the items were normally spoken or melodically intoned, meaningful items showed greater activation in the supramarginal gyrus and inferior parietal lobule, predominantly in the left hemisphere. Melodically intoned language activated both temporal lobes rather symmetrically, as well as the right frontal lobe cortices, indicating that these regions are engaged in the acoustic complexity of melodically intoned stimuli. Compared to spoken language, melodically intoned language activated sensory motor regions and articulatory language networks in the left hemisphere, but only when meaningful language was used.

**Discussion:**

Our results suggest that the facilitatory effect of MIT may – in part – depend on an auditory input which combines melody and meaning.

**Conclusion:**

Combined melody and meaning provide a sound basis for the further investigation of melodic language processing in aphasic patients, and eventually the neurophysiological processes underlying MIT.

## Introduction

Aphasia is a severe language disorder that affects language comprehension and production at different degrees, compromising both spoken and written modalities. The most common cause of aphasia is stroke, in which a neurovascular event damages the language areas localized in the left hemisphere. A common treatment to restore spoken language in severe nonfluent aphasic patients is Melodic Intonation Therapy (MIT) (Albert et al. 1973). This form of therapy has recently received much press attention after the successful recovery of U.S. congresswoman Gabrielle Giffords (Bambury 2011). In a stepwise procedure, MIT uses musical elements of speech such as melody and rhythm (Norton et al. 2009) to help the patient to initiate language production. In the first steps, the speech and language therapist (SLT) shows the patient how to produce a specific target utterance by “singing” the utterance, that is, accentuating its melody and the rhythm. This is accompanied by tapping with the left hand. Such melodically intoned auditory input is thought to play a crucial role in facilitating language production, by priming the patient's inner rehearsal of the target utterance (Norton et al. 2009). MIT's critical elements, intonation, and left-hand tapping, are both thought to be related to right hemisphere activation. Intonation targets the potential role of this hemisphere in processing spectral information, musical features, and prosody, while left-hand tapping engages the right hemisphere sensorimotor network that controls hand and mouth movements (Norton et al. 2009). Although it is not yet clear whether it is melody, rhythm or their combination used in MIT that specifically aid speech production (van der Meulen et al. 2012; Stahl et al. 2013), the treatment has been associated with functional (Vines et al. 2011) and also structural changes in the right hemisphere (Schlaug et al. 2009). The positive effect of this treatment, hypothetically aiding the reorganization of language representation in the damaged brain, has triggered interest in understanding how the musical elements, that are used in MIT, are processed in the brain.

Neuroimaging studies investigating the differences between spoken and melodic language in healthy volunteers have thus far focused primarily on production (i.e. speaking and singing) (Riecker et al. 2000; Jeffries et al. 2003; Ozdemir et al. 2006; Gunji et al. 2007). Despite the methodological diversity of these studies, in general they report a lateralization effect for singing to the right, and speech to the left hemisphere. Thus, encouraging the aphasic patients to use melody during their speech production may target areas in the undamaged right hemisphere, but the question remains what the role is of the melodically intoned auditory input, that is offered intensively during MIT and that probably plays a crucial role in the initial facilitation of language production.

From this point of view, that is, reception instead of production, Meyer et al. (2002) investigated the perceptual differences in processing spoken normal sentences, spoken delexicalized sentences, and prosodic speech (speech utterance reduced to speech melody). Melody (pitch variations in speech) is a component of prosody among several others such as rhythm and loudness (Nooteboom 1997). Their results suggest that right hemispheric activation observed while processing normal speech stimuli mainly comes from the underlying processing of prosody. Later studies have focused on the perception of spoken and sung language, and have shown differences in hemispheric lateralization (Callan et al. 2006; Schön et al. 2010). Speech prosody patterns are similar to the musical features in singing such as melody, rhythm, and loudness, but they exhibit differences regarding their acoustic features. Callan et al. (2006) found right-lateralized activation of the anterior superior temporal gyrus (STG) for sung language, and a strongly left-lateralized activity pattern for spoken language. Schön et al. (2010) suggested that linguistic and musical processing have a different hemispheric specialization. Brain activation patterns for sung versus spoken words showed more extended activations in the right temporal lobe, whereas the processing of linguistic aspects in singing versus vocalization showed a predominance in the left temporal lobe. A recent study of Merrill et al. (2012) found that listening to song and speech activated the temporal lobe rather symmetrically. However, substantial nonoverlap was also found: activation in the inferior frontal gyrus (IFG) was left-lateralized for spoken words as well as for processing pitch in the speech, while right-sided lateralization was found for pitch in the song.

The brain regions involved in the auditory perception of melodically intoned language – a simplified version of singing – have not, to our knowledge, been reported. No more than three to four tones are used to exaggerate speech prosody (Helm-Estabrooks et al. 1989; Sparks 2008). Melodically intoned language is a key feature in MIT and for a greater insight into its neurophysiological processes, this feature needs to be examined. The aim of this study is to investigate the differential perceptual processing of spoken and melodically intoned language using functional MRI. We furthermore assessed whether there was an effect of lexical-semantic content, since it is a meaningful language that MIT uses to improve everyday communication in aphasic patients. A sparse temporal sampling design was employed for acquisition of the functional imaging data to ensure that scanner noise would not interfere with the auditory stimuli, thus being maximally sensitive to differences between the different types of language stimuli.

## Methods

### Participants

Twenty right-handed volunteers (median age: 23 years, range: 21–51 years, 15 females) with no neurological or psychiatric history, participated in this study. None of the participants had any particular musical education. They did not use any prescription medication except oral contraception. Handedness was determined with the Edinburgh Handedness Inventory (Oldfield 1971) indicating 100% right-handedness in all participants. The study was approved by the institutional review board and all participants gave written informed consent prior to participation. Due to technical failure during data acquisition, one participant (female, aged 21 years) was excluded from the analysis.

### Experimental stimuli and paradigm

**Figure 1:**
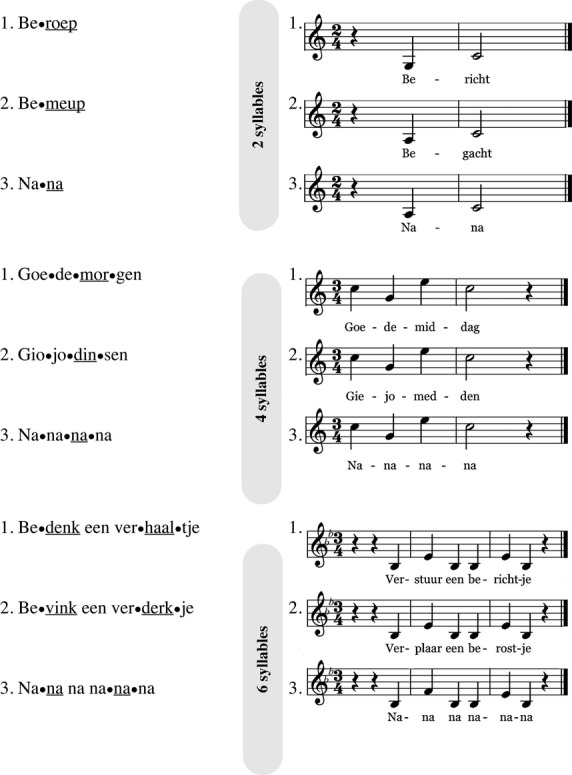
Stimulus examples (in Dutch) of the two experimental conditions. Spoken stimuli (left side of the figure): words are separated into syllables with a black dot. Syllables that are underlined are stressed. Melodically intoned stimuli (right side of the figure): musical notation of the stimulus. In each condition there are three types of stimuli: (1) meaningful, (2) meaningless, and (3) neutral utterances. Provided are examples of words with two and four syllables, and of short phrases of six syllables. Approximately ♩ = 120.

The experiment consisted of two conditions of spoken and melodically intoned stimuli. Each condition contained three categories of 30 items each: (1) 30 *meaningful* items (17 real words and 13 short noun, prepositional or verb phrases); (2) 30 *meaningless* items without lexical-semantic information (17 pseudowords and 13 short phrases containing pseudowords); (3) 30 *neutral utterances*, consisting of a repetitive consonant vocal combination (“Nana”). (Fig. 1; sample stimuli (in Dutch) can be provided upon request). Within and across both conditions, stimuli were matched across the three categories for the number of syllables (range: 2–6), for intonation and stress patterns (for spoken stimuli), melodic contour (for melodically intoned stimuli), semantic content, and syntactic structure of the phrases. We chose to use different words as spoken and melodically intoned stimuli to prevent our participants from becoming familiarized with the words, thus avoiding unwanted and unpredictable effects such as habituation, memory, and learning. Representative examples of the stimuli from both conditions are given in Figure 1, indicating the very minor differences in semantic content between stimuli of a given category such as *“*goede morgen*”* (good morning) in the spoken condition and *“*goede middag*”* (good afternoon) in the melodically intoned condition.

The items were selected by a clinical linguist specialized in MIT and were recorded by a female therapist. Spoken stimuli were recorded with a natural intonation and were not stressed rhythmically in order to keep them as natural as possible. Melodically intoned stimuli were recorded with the same prosodic patterns as those used in MIT. All recorded items had a maximum duration of 3 sec. Melodically intoned items were on average longer than the spoken items (2.24 sec vs. 1.23 sec, respectively; 2-sample t-test *P* < 0.0001).

The experiment was conducted in an event-related design consisting of four experimental conditions and two control conditions. The stimuli in the experimental conditions consisted of 30 melodically intoned meaningful items (“melodic-sense”), 30 spoken meaningful items (“spoken-sense”), 30 melodically intoned meaningless items (“melodic-nonsense”), and 30 spoken meaningless items (“spoken-nonsense”). The two control conditions consisted of the neutral utterances, either melodically –intoned (*n* = 30; “melodic-neutral”) or spoken (*n* = 30; “spoken-neutral”). The task was presented binaurally through an MR compatible headphone system. Participants were required to press the response button upon hearing a meaningful item by pressing the response pad held in the left hand.

Stimuli were pseudo-randomized using the genetic algorithm toolbox Optimize Design 11 (Wager and Nichols 2003) and implemented in Matlab version 6.5.1 (The Mathworks Sherborn, MA), with optimization for the contrast between melodically intoned versus spoken language primarily (which we will refer to as acoustic information), and for the contrast between meaningful and meaningless language secondarily (lexical-semantic information).

The task was presented using Presentation v13.0 software (Neurobehavioral Systems Inc. Albany, CA) installed on a desktop PC, which was dedicated for stimulus presentation. External triggering by the MR system ensured synchronization of the stimulus paradigm with the imaging data acquisition and precise recording of task performance, and response times through a fiber-optic button response pad.

Participants were familiarized with the task prior to scanning with a sample set of representative items. Behavioral data (responses and reaction times) were collected during scanning. Differences in performance between melodically intoned and spoken items were assessed with a two sample *t*-test.

### fMRI image analysis

#### Imaging acquisition and preprocessing

Scanning was performed on a 3T MR system (HD platform, GE Healthcare, Milwaukee, WI). An 8-channel head coil was used for reception of the signal.

For anatomical reference, a high-resolution 3 dimensional (3D) Inversion Recovery (IR) Fast Spoiled Gradient Echo (FSPGR) T1-weighed sequence was used, with the following pulse sequence parameters: repetition time (TR)/echo time (TE)/inversion time (TI) 10.5/2.1/300 ms; flip angle 18°; acquisition matrix 416 × 256; field of view (FOV) 250 × 175 mm^2^; 172 slices with a slice thickness of 1.6 mm and 0.8 mm overlap; acquisition time 4:40 min.

For functional imaging, a sparse temporal sampling design was employed for acquisition of the functional imaging data, using a single shot T2*-weighted gradient echo echo-planar imaging (EPI) sequence sensitive to blood oxygenation level dependent (BOLD) contrast (TE 30 ms; flip angle 75°; acquisition matrix 64 × 96; FOV 220 × 220 mm^2^; slice thickness 3.5 mm with no gap; 39 slices with full brain coverage). TR was 6000 ms and acquisition time 3000 ms resulting in a 3000 ms silent gap which was used for presentation of the auditory stimulus. Total duration was 18:30 min.

The functional imaging data acquisition included five dummy scans that were discarded from further analysis. Imaging analysis was performed using SPM8 (Statistical Parametric Mapping; Wellcome Trust Centre for Neuroimaging, London, UK). Images were manually reoriented to the anterior commissure and subsequently all T2*-weighed functional images were realigned to correct for the participant's motion during data acquisition and were coregistered with the individual's high-resolution T1-weighed anatomical image (Friston et al. 1995). The functional and anatomical images were normalized to the standard brain space defined by the Montreal Neurological Institute (MNI) as provided within SPM8, using affine and nonlinear registration. This resulted in resampled voxel sizes of 3 × 3×3 mm^3^ for the functional and 1 × 1×1 mm^3^ for the anatomical images. The normalized functional images were smoothed with a 3D Gaussian Full Width Half Maximum (FWHM) filter of 6 × 6×6 mm^3^ to increase the signal-to-noise ratio, correct for interindividual anatomical variation and to normalize the data (Friston et al. 1999).

#### Statistical analysis of fMRI data

All fMRI data were analyzed within the context of the General Linear Model (GLM), by modeling the experimental conditions convolved with the hemodynamic response function (HRF), corrected for temporal autocorrelation and filtered with a high-pass filter of 128 sec cutoff. The neutral conditions were not modeled and served as an implicit baseline. To account for the sparse sampling acquisition, we defined the micro time resolution and onset based on the time bin that corresponded to the middle of the actual acquisition time (1500 ms). Motion parameters were included in the model as regressors of no interest to reduce the potential confounding effects due to motion. Because of the significantly longer duration of the melodically intoned versus the spoken stimuli, stimulus duration was modeled as an additional regressor of no interest to account for confounding stimulus duration effects. The individual t-contrast images for spoken-sense, spoken-nonsense, melodic-sense, and melodic-nonsense were used to perform a full-factorial ANOVA group analysis (*n* = 19 participants). The two within-subject factors, prosody and lexical-semantic information (equal variance, levels not independent), were entered in this analysis. Main effects as well as the interaction between these factors were investigated. The following contrasts were created to evaluate the main effects of lexical-semantic information: sense > nonsense and nonsense > sense; and of acoustic information: spoken > melodic and melodic > spoken. Interaction effects for acoustic information with lexical-semantic information were explored with the following contrasts: spoken-sense versus spoken-nonsense, melodic-sense versus melodic-nonsense, spoken-sense versus melodic-sense, and spoken-nonsense versus melodic-nonsense. The threshold for significance was set at *P* < 0.05 family wise error (FWE) corrected for multiple comparisons.

Anatomical labeling of significantly activated clusters was performed using the Automated Anatomical Labeling map (Tzourio-Mazoyer et al. 2002) software extension to SPM8, using the extended local maxima labeling option. Figures were created with the SPM render function.

## Results

### Task performance

Participants performed well in both conditions with an average accuracy of 96% (SD: 3%). Performance was equally high in both conditions (*P* = 0.486).

### fMRI activation results

#### Lexical-semantic information: main effect and interactions

**Figure 2:**
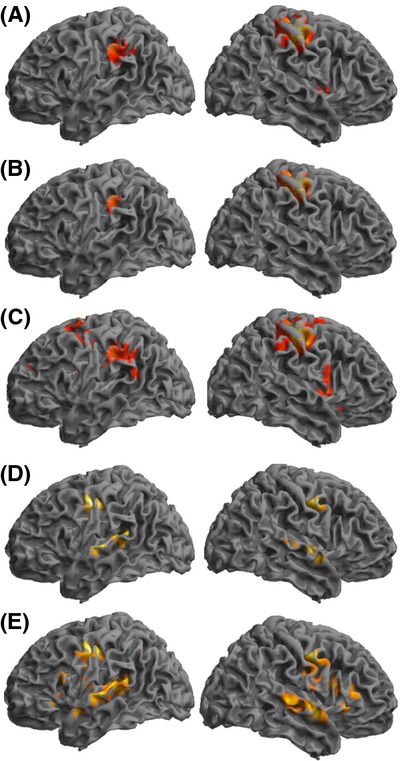
Three dimensional brain rendering with superposition of the activation maps displayed at *P*FWE corrected<0.05, *k* ≥ 10 for the following contrasts: (A) sense > nonsense stimuli, (B) spoken-sense > spokennonsense stimuli, (C) melodic-sense > melodic-nonsense, (D) melodic > spoken stimuli, (E) melodicsense > spoken- sense stimuli.

We found a main effect for the lexical-semantic information factor (F (1,72) = 26.27 *P*_FWE corrected_ <0.05). Post hoc analysis revealed no increased activation for the meaningless items compared to meaningful items (nonsense > sense). For the meaningful items compared to meaningless items (sense > nonsense) increased activation was seen left-lateralized in the supramarginal gyrus (SMG) and inferior parietal lobule (IPL). Increased bilateral activation was seen in the rolandic operculum, insula, supplementary, and cingulate motor area. Right-sided activation was observed in the pre- and postcentral gyrus at the level of the hand motor area, presumably due to the button presses (Fig. 2A; Table 1).

**Table 1:** Anatomical location, cluster sizes (*k*, number of voxels), MNI coordinates, and statistical *T*-values of areas of significant activation for the contrast sense > nonsense (*P*_FWE corrected_ < 0.05, *k* ≥ 10). The percentages reflect the proportion of the activated cluster localized in each anatomical region

Anatomical location	Side	Cluster size	MNI			*T*-value

			*x*	*y*	*z*	
Inferior parietal lobule (50%)	L	259	−54	−31	40	8.08
Supramarginal gyrus (40%)	L					
Rolandic operculum/insula (100%)	L	24	−48	−1	4	5.87
Rolandic operculum/insula (100%)	R	34	48	5	4	6.27
Supplementary motor area (70%)	L/R	512	6	−4	52	10.00
Middle cingulate gyrus (50%)	L/R					
Pre- and postcentral gyrus (82%)	R	645	36	−22	49	15.57
Supramarginal gyrus (5%)	R					
Inferior parietal lobule (4%)	R					
Thalamus (50%)	R	51	15	−22	4	6.51
Cerebellum (100%)	L	23	−18	−61	−23	5.74

L, left hemisphere; R, right hemisphere; MNI, Montreal Neurological Institute.

For spoken items, no significantly increased activation was found for meaningless compared to meaningful items (spoken-nonsense > spoken-sense). However, increased activation was seen for meaningful compared to meaningless items (spoken-sense > spoken-nonsense) in the left SMG and IPL, and bilaterally in the supplementary and cingulate motor area (Fig. 2B; Table 2). Furthermore, there was increased right-sided activation in the pre- and postcentral gyrus, presumably due to the button presses.

**Table 2:** Anatomical, cluster sizes (*k*, number of voxels), MNI coordinates, and statistical *T*-values of areas of significant activation for the contrast spoken-sense > spoken-nonsense (*P*_FWE corrected_ < 0.05, *k* ≥ 10). The percentages reflect the proportion of the activated cluster localized in each anatomical region

Anatomical location	Side	Cluster size	MNI			T-value

			*x*	*y*	*z*	
Inferior parietal lobule (57%)	L	63	−54	−31	40	6.82
Supramarginal gyrus (43%)	L					
Supplementary motor area (70%)	L/R	147	6	−7	52	7.77
Middle cingulate gyrus (30%)	L/R					
Pre- and postcentral gyrus (94%)	R	395	42	−25	55	12.91

L, left hemisphere; R, right hemisphere; MNI, Montreal Neurological Institute.

For melodically intoned items, no significantly increased activation was found for melodically intoned meaningless compared to meaningful items (melodic-nonsense >melodic-sense). For meaningful items compared to meaningless items (melodic-sense > melodic-nonsense) increased activation was seen left-lateralized in the SMG and IPL. Left-sided activation was observed in the posterior portion of the middle and superior temporal gyrus (Sylvian parieto-temporal area) and in the middle and superior frontal gyrus (Fig. 2C; Table 3). Right-lateralized activation was seen in the insula, rolandic operculum, and pars opercularis of the inferior frontal gyrus (IFG). Increased bilateral activation was observed in the supplementary and cingulate motor area. Furthermore, increased right-lateralized activation in the pre- and postcentral gyrus was seen, presumably due to the button presses.

**Table 3:** Anatomical, cluster sizes (*k*, number of voxels), MNI coordinates, and statistical *T*-values of areas of significant activation for the contrast melodic-sense > melodic-nonsense (*P*_FWE corrected_ < 0.05, *k* ≥ 10). The percentages reflect the proportion of the activated cluster localized in each anatomical region

Anatomical location	Side	Cluster size	MNI			*T*-value

			*x*	*y*	*z*	
Inferior parietal lobule (50%)	L	293	−51	−31	37	6.94
Supramarginal gyrus (40%)	L					
Inferior parietal lobule (20%)	L	27	−30	−73	40	6.32
Angular gyrus (5%)	L					
Occipital middle gyrus (75%)	L					
Superior and middle temporal gyrus (100%)	L	37	−57	−52	19	6.39
Superior and middle frontal gyrus (100%)	L	10	−21	20	58	5.91
Middle frontal gyrus (90%)	L	28	−30	35	25	5.89
Inferior frontal gyrus: pars triangularis (10%)	L					
Insula (85%)	L	21	−36	11	4	5.70
Rolandic operculum/insula (97%)	L	24	−40	−1	7	5.75
Rolandic operculum/insula (66%)	R	146	48	5	1	7.34
Inferior frontal gyrus: pars opercularis (10%)	R					
Supplementary motor area (37%)	L/R	900	6	−4	52	9.37
Middle cingulate gyrus (40%)	L/R					
Pre- and postcentral gyrus (75%)	L	20	−54	2	22	5.58
Pre- and postcentral gyrus (77%)	R	669	36	−22	49	13.81
Supramarginal gyrus (7%)	R					
Inferior parietal lobule (4%)	R					
Thalamus (100%)	L	16	−12	−28	10	5.59
Thalamus (39%)	R	122	−3	−25	−2	7.01
Putamen (85%)	R	13	21	17	−11	5.35
Cerebellum (100%)	L	36	−21	−61	−23	5.95

L, left hemisphere; R, right hemisphere; MNI, Montreal Neurological Institute.

#### Acoustic information: main effect and interactions

We found a main effect for the acoustic information factor (*F*(1,72) = 26.31 *P*_FWE corrected_ <0.05). Post hoc analysis revealed no increased activation for spoken compared with melodically intonated items (spoken > melodic). For the melodically intoned compared to spoken items (melodic > spoken), increased activation was seen bilaterally, but more pronounced in the left hemisphere, in the superior and middle temporal gyrus, Heschl's gyrus, supplementary motor area, and in the ventral pre- and postcentral gyrus (at the level of the primary motor and somatosensory area of the face). In the posterior portion of the superior and middle temporal gyrus, (Sylvian parieto-temporal area) activation was mainly left sided (Fig. 2D; Table 4).

**Table 4:** Anatomical location, cluster sizes (*k*, number of voxels), MNI coordinates, and statistical *T*-values of areas of significant activation for the contrast melodic > spoken (*P*_FWE corrected_ < 0.05, *k* ≥ 10). The percentages reflect the proportion of the activated cluster localized in each anatomical region

Anatomical location	Side	Cluster size	MNI			*T*-value

			*x*	*y*	*z*	
Superior and middle temporal gyrus (88%)	L	60	−51	−16	4	8.79
Heschl's gyrus (12%)	L					
Superior and middle temporal gyrus (75%)	L	92	−51	−40	13	7.74
Heschl's gyrus (4%)	L					
Superior temporal gyrus and pole (92%)	R	76	54	−10	1	7.16
Heschl's gyrus (7%)	R					
Superior temporal gyrus (100%)	R	12	66	−26	7	5.63
Supplementary motor area (100%)	L/R	45	−3	−1	64	7.06
Pre- and postcentral gyrus (100%)	L	68	−51	−13	43	8.93
Pre- and postcentral gyrus (100%)	R	41	54	−4	43	7.72

L, left hemisphere; R, right hemisphere; MNI, Montreal Neurological Institute.

For meaningless items, no increased activation was found for spoken versus melodically intoned items (spoken-nonsense > melodic-nonsense; melodic-nonsense > spoken-nonsense). Furthermore, for meaningful items, no increased activation was found for spoken compared with melodically intoned meaningful items (spoken-sense > melodic-sense). Only for melodically intoned compared to spoken meaningful items (melodic-sense > spoken-sense) increased activation was seen bilaterally in the superior and middle temporal gyrus, insula, supplementary and cingulate motor area, and in the ventral pre- and postcentral gyrus (at the level of the primary motor and somatosensory area of the face). Right-lateralized activation was seen in the pars opercularis and triangularis of the IFG. Left-sided activation was seen in the posterior portion of superior and middle temporal gyrus (Sylvian parieto-temporal area) (Fig. 2E; Table 5).

**Table 5:** Anatomical, cluster sizes (*k*, number of voxels), MNI coordinates, and statistical *T*-values of areas of significant activation for the contrast melodic-sense > spoken-sense (*P*_FWE corrected_ < 0.05, *k* ≥ 10). The percentages reflect the proportion of the activated cluster localized in each anatomical region

Anatomical location	Side	Cluster size	MNI			*T*-value

			*x*	*y*	*z*	
Superior and middle temporal gyrus (48%)	L	578	−51	−13	43	9.73
Heschl's gyrus (5%)	L					
Pre- and postcentral gyrus (36%)	L					
Superior and middle temporal gyrus (100%)	L	25	−51	−1	−11	6.44
Superior and middle temporal gyrus (90%)	R	315	54	−10	−2	7.59
Heschl's gyrus (6%)	R					
Superior temporal pole (4%)	R					
Angular gyrus (29%)	R	17	33	−64	34	5.62
Superior and middle occipital gyrus (71%)	R					
Insula (57%)	L	19	−27	23	−2	6.13
Insula (48%)	R	25	30	23	−2	5.89
Inferior frontal gyrus pars opercularis (80%)	L	38	−45	14	19	6.38
Inferior frontal gyrus pars triangularis (20%)	L					
Inferior frontal gyrus pars triangularis (25%)	R	271	54	−4	43	7.83
Inferior frontal gyrus pars opercularis (18%)	R					
Pre-and postcentral gyrus (46%)	R					
Supplementary motor area (51%)	L/R	282	−6	2	61	7.60
Superior medial frontal gyrus (30%)	L/R					
Middle cingulate gyrus (10%)	R					
Caudate nucleus (100%)	R	28	9	11	1	5.86

L, left hemisphere; R, right hemisphere; MNI, Montreal Neurological Institute.

## Discussion

Using a dedicated silent-gap acquisition, we found different patterns of activation for the auditory processing of melodically intoned language compared to normal spoken language. Compared to spoken language, melodic language recruited left-sided brain regions in the left posterior portion of the superior and middle temporal gyrus (Sylvian parieto-temporal area), as well as the operculum and IFG with a right-sided lateralization. Additionally, there was activation along the superior temporal gyrus bilaterally. With regards to lexical-semantic processing, spoken and melodically intoned language showed similar left-sided activation in the SMG and IPL.

Although our primary focus was to investigate auditory perception of spoken and melodically intoned language, we also investigated the informative content of the auditory stimuli. In the context of MIT this is important, because patients are trained with meaningful items, initially those that are frequently used in everyday language and then progressing to less familiar utterances. The selected meaningful (real words) and meaningless (pseudowords) items only differed with respect to their accessibility to lexical access and meaning. For meaningful items both the word form and lexical-semantic content are successfully accessed, while such information is not available for meaningless items. We did not find any increased activation for meaningless compared to meaningful language. This finding is in line with the results of Binder et al. (2000) who also did not find differences when directly comparing brain activation patterns of participants passively listening to meaningless words (pseudowords and reversed words) with meaningful words. Furthermore, our results showed that irrespective of whether the items were normally spoken or melodically intoned, meaningful items showed greater activation in the SMG and IPL. This is in line with a review by Fiez (1997) who suggested that long-term storage of conceptual and semantic knowledge is dependent on posterior regions (Fiez 1997). As expected, this activation was lateralized to the left hemisphere, which is dominant for speech processing (Knecht et al. 2000; Tallal 2012). This finding is generally aligned with previous neuroimaging studies investigating lexical-semantic processing which, despite the use of various different tasks designs, reported activation for meaningful language in the inferior parietal areas around the temporo-parietal junction (Price 2000; Kotz et al. 2002; Vigneau et al. 2005; Xiao et al. 2005). The activation emerging from such lexical decision tasks can principally be attributed to either lexical access or semantic processing. Contrary to what lesion language models propose, these two main processes are difficult to disentangle in the undamaged brain.

Overall, melodically intoned stimuli compared to spoken stimuli showed bilateral, somewhat left-lateralized activation, in the superior temporal gyrus and frontal/motor regions. Left-sided activation was seen in the posterior portion of the superior and middle temporal gyrus, which was coined by Hickok and Poeppel (2000) the Sylvian parieto-temporal (Spt) area. This Spt area is thought to be a part of an auditory motor integration system: a sensorimotor interface related to both speech comprehension and phonological aspects of speech production (Buchsbaum et al. 2001; Hickok et al. 2003, 2009). This area is thus activated for language production and guides speech perception. Nevertheless, Hickok et al. (2003) suggested that activation in the Spt area is not specifically dedicated to speech because it was found to be equally activated by both speech and nonspeech stimuli. In fact, the Spt area was even found to respond better to music stimuli than to speech, indicating some degree of specificity for tonal stimuli within portions of this area. This degree of specificity for tonal stimuli is in line with our results showing increased activation for melodically intoned items, presumably due the tonal pattern of the melodic stimuli. So although this area is maybe not unique to speech signals as suggested by Hickok et al. (2003) it is sensitive to the tonal differences between normal speech and melodically intoned speech. What is interesting to note, however, is that we found pronounced activation in the Spt area specifically for the processing of *meaningful* melodically intoned items. Thus, it is not only the tonal pattern that triggers the activation in this area, but it is also the lexicality of the stimuli that plays an important role in activating this area.

The activation in the Spt area was accompanied by bilateral ventral motor activation at the level representing the face, and there was an additional activation in the left IFG when lexical-semantic content was present. These findings can partially be interpreted in the context of the dorsal stream model proposed by Hickok and Poeppel (2007) for auditory processing. The dorsal stream projects connections from the Spt area to the left frontal cortices, specifically to the dorsal portion of the premotor cortex and to the left IFG and ventral portion of the premotor cortex. The latter two are called the articulatory network (Hickok and Poeppel 2007). This stream is thought to be involved in translating acoustic speech signals into articulatory representations in the frontal lobe. It is essential for speech production and guides speech perception before the next stage of speech comprehension (Hickok and Poeppel 2007). Furthermore, the bilateral activation in the primary motor area at the level representing the face may be interpreted in the context of the pioneer motor theory of speech perception proposed by Liberman and Mattingly (1985). This theory suggests that coarticulation occurs in parallel to auditory processing to aid the auditory system in separating speech segments over longer intervals of time (Kotz et al. 2010). Taken together, our findings suggest that melodically intoned language perception recruits the articulatory system in the dorsal stream as well as motor priming areas more strongly than that of spoken language. This is an important finding in the context of MIT, since the first stages of this therapy focus on intensively providing auditory input with prosodic features different from those used in normal speech. Such auditory input, simulated here with melodically intoned speech items, thus hypothetically serves to facilitate the activation of the articulatory system and priming of the motor areas for language production. Again, it seems that lexical-semantic content needs to be present for such processes to be optimally involved.

Furthermore, melodically intoned stimuli activated both temporal lobes rather symmetrically, as well as the right frontal lobe cortices, more than the normally spoken stimuli. This finding is in line with the study of Merrill et al. (2012). By using both a univariate and multivariate analysis, the authors identified overlapping activation for song and spoken language in the superior temporal lobe bilaterally, but also suggested a differential role of the IFG and intraparietal sulcus in processing song and speech. Similar overlapping activation for speech and music stimuli in the superior temporal lobe bilaterally has been reported by Rogalsky et al. (2011). In a review of fMRI studies investigating language processing, Price (2010) highlighted that bilateral superior temporal lobe activation likely reflects differences in the acoustic complexity of the presented auditory stimuli. The present findings are, therefore, most likely a reflection of the different levels of auditory processing within the auditory cortex involved with melodically intoned language. We found that there was no increased activation along the superior temporal lobe during the auditory processing of spoken compared with melodically intoned stimuli, suggesting that the superior temporal lobe activation likely reflects the processing of different temporal information present in melodic intonation due to longer syllable duration (Zatorre and Belin 2001). This is a feature that aphasic patients following MIT may also get benefit from, because they also have a basic deficit processing the rapidly changing sequential information (Tallal and Newcombe 1978). In addition, we see that the right frontal operculum and the pars opercularis of the IFG are more engaged in the processing of melodically intoned compared with spoken stimuli. The study of Merrill et al. (2012) reported a similar role of the right IFG for pitch processing in song. Similar results were previously reported by Meyer et al. (2002), who investigated brain activation of the prosodic patterns of normal speech. This finding supports in part the hypothesis underlying MIT that musical elements of speech (melody and rhythm) engage right hemisphere frontal cortices. In melodically intoned language, which is a simplified version of singing, speech prosodic patterns are exaggerated by altering many acoustic features of normal spoken language (Belin et al. 1996). The type of prosody we use in our melodically intoned stimuli is referred to as linguistic prosody, a type of prosody used in normal speech when stressing syllables, changing intonation while asking a question, and even when using intentioned melodies during mother-to-child speech. It is indeed the pars opercularis of the IFG, according to a recent meta-analysis of Belyk and Brown (2013) that is more likely to become active with linguistic prosody.

Some neuroimaging studies have aimed to differentiate the neural mechanisms of musical features of speech by either comparing spoken language with sung language or by using novel tones. To our knowledge, no previous neuroimaging study has investigated the neural processing of melodically intoned meaningful language, an essential feature of MIT. While our findings strongly support the hypothesis that melodically intoned language is processed differently from spoken language, there are some issues that may need to be taken into account. Firstly, in order to keep participants engaged during the experiment, we decided to include a button press. The hand motor activation could easily be identified and could, therefore, simply be disregarded to not interfere with the further interpretation of the results of interest. Nevertheless, we need to consider the possibility that this button press upon meaningful words may have shifted attention toward meaningful items. Secondly, melodically intoned language is inherently slower than spoken language. The consequently longer exposure to melodically intoned stimuli may lead to unspecific increases in activation, which we accounted for by modeling the stimulus duration as a regressor of no interest. Thirdly, our stimuli set included both words and short phrases, so some confounding of lexical-semantic and syntacting processing cannot be excluded with certainty. Finally, and crucially, although our eventual interest is aimed at understanding the effect of melody used in MIT for the treatment of aphasic patients, here we investigated the processing of melodic language in healthy participants. This is the first and necessary step in understanding the neurophysiological mechanisms underlying MIT, but our findings cannot be directly translated to aphasic patients. In our future work we will investigate melodic language processing, as well as the effect of MIT, in aphasic patients.

In conclusion, this study demonstrates that the auditory processing of melodically intoned language activates a left-lateralized motor-sensory network, which is much more engaged when lexical-semantic content is present, related to the articulatory system and motor priming. These systems are of great interest in the context of MIT. In line with the observations from lesion studies, Belin et al. 1996; that perilesional activation appears in aphasic patients after successful MIT, we can hypothesize that this therapy triggers not only activation in areas in the right hemisphere (as it was initially hypothesized by the developers of MIT), but may also activate perilesional areas in the left hemisphere. Naeser and Helm-Estabrooks (1985), reported that patients with a lesion in Broca's area that extended to pre-motor area and lower motor-sensory cortex area of the face are those that benefit the most of MIT therapy. When using the MIT technique, SLTs provide the aphasic patient with an auditory input of melodically intoned meaningful language. This activation might facilitate the production of the primed utterances, which enables the patient to train production of meaningful utterances. In addition, we found right hemispheric activation in the frontal operculum and IFG, which supports in part the hypothesis underlying MIT that musical elements of speech (melody) engage right hemisphere frontal cortices. The combination of melody and meaning in the auditory input may be a crucial aspect of MIT and that this technique improves language production by targeting language function as well as speech functions. Our current study provides a sound basis for the further investigation of melodic language processing in aphasic patients, and eventually the neurophysiological processes underlying MIT.
